# Special Issue on “Qualitative Inquiry in Mental Health Research with Young People”

**DOI:** 10.3390/ijerph18126660

**Published:** 2021-06-21

**Authors:** Katherine M. Boydell, Michael Hodgins

**Affiliations:** 1 Black Dog Institute, School of Psychiatry, University of New South Wales, Randwick, NSW 2031, Australia; 2 School of Women’s and Children’s Health, University of New South Wales, Randwick, NSW 2031, Australia; michael.hodgins1@unsw.edu.au

**Keywords:** qualitative inquiry, mental health, young people

## Abstract

This editorial to the Special Issue on Qualitative Inquiry in Mental Health Research with Young People provides an overview of the importance of qualitative inquiry to the field of child and youth mental health. The issue highlights research using qualitative methods to depict the lived experiences and contribution of young people in areas that reflect important mental health concerns, ranging from anxiety, non-suicidal self-injury, positive resilience in young people in times of crisis, and drug and alcohol treatment.

## 1. Introduction

Qualitative inquiry provides an opportunity for research participants to offer authentic experiential evidence to help shape policy and mental health service provision from an evidence-based perspective. Qualitative methods provide richly textured, thick description to supplement the breadth of understanding afforded by quantitative methods, elicit the perspectives and lived experience of research participants, explore issues that have not been well studied, develop conceptual theories or test hypotheses, or examine a phenomenon or intervention.

This Special Issue aims to discuss new knowledge and cutting-edge developments in qualitative mental health research with young people through selected works, each making an important contribution to the extant knowledge base. It represents a forum for disseminating pertinent research findings and innovative ideas and methods from the field.

Young people are rarely meaningfully involved in contributing to the development, implementation, and evaluation of interventions designed to address their mental health challenges [1]. However, qualitative methods, which are increasingly being taken up in the mental health arena [2], are well suited to research with young people. They offer the opportunity for young people to express their perspectives and share their experiences using strategies that encourage meaningful involvement [3]. The most common techniques in the qualitative researcher’s toolbox are individual semi-structured interviews, focus groups, document reviews, and participant observation. Recent work has introduced arts-based qualitative methods such as photovoice [4], digital storytelling [5] and body mapping [6]. The application of such qualitative methods is broad, ranging from exploratory work in generating knowledge, building theory and conceptual frameworks to understanding the phenomena under study, and influencing practice and policy change [7]. Qualitative methods are generally integral to youth-centred, participatory approaches that privilege the experiences and perspectives of participants in research.

## 2. The Special Issue

This Special Issue highlights research using qualitative methods to depict the lived experiences and contribution of young people in areas that reflect important mental health concerns, ranging from non-suicidal self-injury, positive resilience in young people in times of crisis, and drug and alcohol treatment.

The contributors to this Special Issue drew from a range of methodological perspectives and employed a variety of qualitative data collection methods, including arts-based methods, such as body mapping, photovoice and ecomaps, secondary documents, in-depth interviews, participant observation and journaling. The contributors also drew upon diverse forms of analysis including hermeneutic phenomenology, reflexive thematic analysis, case study, and narrative analysis. The studies described were conducted in Canada, Europe (UK, Germany, Austria), and Australia.

Cus et al. [8] use reflexive thematic analysis to qualitatively assess user needs and preferences of 15 young women (12–18 years) who engaged in non-suicidal self-injury regarding smartphone mental health interventions. Their goal was to develop a framework for engaging digital resources for mental health and to generate ideas on how future mental health interventions could potentially be more engaging. Young people’s responses are organised along three different contexts: the mental health condition, the person using the intervention and the technology. Identified preferences were for a more flexible user-driven approach, support for young people in moments of crisis when they experience the urge to self-injure, content that is adaptive to everyday life settings such as home and school, and receipt of support at different times.

COVID-19 has been associated with loneliness, distress, and anxiety in young people. In Dadich and colleagues’ [9] methodological article, exemplars sourced from social media platforms are used to highlight the creative brilliance of young people during these times of crisis. These secondary sources reflect how the arts can be used to highlight positive experiences during crisis and learn from the creative brilliance of young people. The authors posit that positive organisational arts-based youth scholarship has utility for understanding the ways in which young people use the arts to redress negativity and focus on agency, peace, and collectedness. Positive organisational scholarship allows for the examination, understanding and promotion of experiences that generate positive emotion and bolster resilience.

Francis and her colleagues [10] investigated parent and pre-adolescent perspectives regarding screen use and the source of conflict around social media in a small town in Ontario, Canada. Using a qualitative analysis of naturalistic conversation, they found that parents and children have conflicts regarding screens, hold different perspectives, and place different values on screen use. Young people were willing to compromise about screen use with their parents and recognised the potential costs and benefits of screen use. The authors conclude that social messaging and media discussion about screen use needs to change.

The methodological article by Macken and her colleagues [11] explores the utility of using body mapping, an arts-based method, in the drug and alcohol treatment sector in Sydney, Australia, to explore young people’s strengths and support networks. They found body mapping facilitated discussions on subject matter normally challenging for young people to talk about. It engaged and focused young people on a task and created a platform for the expression of strengths and supports. The creative process of crafting a body map encouraged reflection, allowed ownership of the story conveyed, and generated a feeling of pride and accomplishment. Visual methods such as body mapping hold promise for working with young participants in alcohol and other drug treatment and other research settings as an engaging and less confrontational form of data collection.

The service experiences of young people and their parents/carers who completed Dialectical Behaviour Therapy (DBT) treatment in the UK were explored by Ratnaweera and co-authors [12]. Young people indicated that the treatment program was experienced as a person-centred approach, and resulted in a new way of living, better self-understanding, and the acquisition of new skills. Parents/carers expressed their experience of improved relationships, feeling supported, and enhanced quality of life.

Samir and colleagues [13] acknowledged the dearth of research that involves young people from culturally and linguistically diverse communities in the entire research process, including design, analysis, and dissemination. Their health priority mapping work with a Youth Advisory Group in South West Sydney revealed that their main areas of concern when thinking about health were mental health and stress. The research team highlight the bottom-up approach they used to meaningfully involve young students in the process; for example, using young people as workshop facilitators.

Woodgate and her colleagues [14] sought to highlight the voice of young people in Winnipeg, Canada, living with anxiety. They drew upon in-depth interviews, photovoice and ecomaps. Anxiety was described by youth as a ‘monster’—with associated feelings of fear, loss and pain, but also hope. This represents the first study to reveal that young people use metaphors to make sense of living with anxiety; they provide insights into the broader views of their lived space, lived body, lived time and lived relationships. The authors conclude by arguing for the use of narrative and visual metaphors as a communicative strategy to convey the experiences of young people to researchers, clinicians, and the public.

## 3. Conclusions

This Special Issue provides a broad overview of established and emerging qualitative methodological approaches and methods used to involve young people in mental health research. It represents the richness and strength of the field currently, which will no doubt continue to contribute mental health outcomes for young people moving forward.

## Data Availability

Not applicable.
